# DNA from extinct giant lemurs links archaeolemurids to extant indriids

**DOI:** 10.1186/1471-2148-8-121

**Published:** 2008-04-28

**Authors:** Ludovic Orlando, Sébastien Calvignac, Céline Schnebelen, Christophe J Douady, Laurie R Godfrey, Catherine Hänni

**Affiliations:** 1 Paléogénétique et Evolution Moléculaire, Université de Lyon, Institut de Génomique Fonctionnelle de Lyon, Institut Fédératif Biosciences Gerland Lyon Sud, Université Lyon 1, CNRS, INRA, Ecole Normale Supérieure de Lyon, 46 allée d'Italie, 69364 Lyon Cédex 07, France; 2 CNRS UMR 5023, Laboratoire d'Ecologie des Hydrosystèmes Fluviaux, Université Claude Bernard Lyon 1, 6 rue R. Dubois, Bat. Darwin-C, F-69622 Villeurbanne Cédex, France; 3 Department of Anthropology, 240 Hicks Way, University of Massachusetts, Amherst, MA 01003, USA

## Abstract

**Background:**

Although today 15% of living primates are endemic to Madagascar, their diversity was even greater in the recent past since dozens of extinct species have been recovered from Holocene excavation sites. Among them were the so-called "giant lemurs" some of which weighed up to 160 kg. Although extensively studied, the phylogenetic relationships between extinct and extant lemurs are still difficult to decipher, mainly due to morphological specializations that reflect ecology more than phylogeny, resulting in rampant homoplasy.

**Results:**

Ancient DNA recovered from subfossils recently supported a sister relationship between giant "sloth" lemurs and extant indriids and helped to revise the phylogenetic position of *Megaladapis edwardsi *among lemuriformes, but several taxa – such as the Archaeolemuridae – still await analysis. We therefore used ancient DNA technology to address the phylogenetic status of the two archaeolemurid genera (*Archaeolemur *and *Hadropithecus*). Despite poor DNA preservation conditions in subtropical environments, we managed to recover 94- to 539-bp sequences for two mitochondrial genes among 5 subfossil samples.

**Conclusion:**

This new sequence information provides evidence for the proximity of *Archaeolemur *and *Hadropithecus *to extant indriids, in agreement with earlier assessments of their taxonomic status (Primates, Indrioidea) and in contrast to recent suggestions of a closer relationship to the Lemuridae made on the basis of analyses of dental developmental and postcranial characters. These data provide new insights into the evolution of the locomotor apparatus among lemurids and indriids.

## Background

At the time of the first settlers over 2000 years ago [1,2], Madagascar harboured a greater faunal and floral diversity than today. Pygmy hippos and the world's largest bird – the Great Elephant Bird *Aepyornis maximus *– are just two striking examples of those endemic species that began to decline and finally disappeared completely in the centuries following human colonization [1,3]. Albeit emblematic of Madagascar (>90 species among 15 genera currently alive in Madagascar, which represents about 15% of the whole diversity among Primates), lemurs do not stand as an exception [4-6]. Some 2,000 years ago, they inhabited a wide variety of wooded terrains, from forests to open woodlands and marshlands [7]. A spectacular array of life history traits derives from a single ancestral primate that colonized Madagascar around 60MYA [8]. But human activities, such as overhunting and habitat modifications related to farming and pastoralism, led at least 17 species – belonging to nine different genera – to eventual extinction [9,10]. Several entire families – the Archaeolemuridae, Palaeopropithecidae, and Megaladapidae – disappeared.

Because all of the extinct species were larger than extant species, they are called 'giant' lemurs. Some of them displayed particularly spectacular features (for example, enormous body size, elongated rostra and widely separated orbits, extremely rapid dental development). The Palaeopropithecidae, including the most specialized genus, *Palaeopropithecus *and its close relative *Archaeoindris*, as well as the smaller-bodied *Babakotia *and *Mesopropithecus*, were so convergent on tree sloths that they have been dubbed the "sloth lemurs" [11]. However, *Archaeoindris*, despite its specializations for climbing, would have had to spend most of its time on the ground due to a body mass (ca. 160 kg) rivalling that of male gorillas [12]. The megaladapids, some of which rivalled female gorillas in body mass at ca. 88 kg [12], were slow climbers capable of suspension but not leaping [13]. They sported peculiar cranial specializations for consuming leaves and converged postcranially with koalas; they have thus been dubbed "koala lemurs."

Extinct and extant lemurs exhibit an extraordinary range in body size and diversity of locomotor and dietary patterns. Ecologically driven convergences have considerably confounded interpretations of the phylogenetic relationships among lemurs. However, morphological data (cranial and postcranial characters, developmental features) have aided in the construction of a number of different phylogenetic hypotheses [14,15] some of which have been tested using molecular tools. For instance, Crovella *et al*. (1994) used hybridization features of highly repeated DNA probes to support the proximity of the extinct *Pachylemur insignis *to the ruffed lemurs (*Varecia variegata*) [16]. More recently, using PCR to recover short overlapping fragment over the cytochrome *b *gene, Yoder *et al*. (1999) [17] and Karanth *et al*. (2005) [18] were able to confirm Palaeopropithecidae as a sister group of extant indriids but challenged the long-standing proximity of *Megaladapis *to *Lepilemur*.

Other thorny phylogenetic questions still await a molecular contribution. Such is the case for the least arboreal and most terrestrial lemurs, the Archaeolemuridae [19,20]. Among Lemuriformes, this family is presumed to belong to the superfamily Indrioidea and consists of three extinct species: *Archaeolemur edwardsi*, *Archaeolemur majori*, and *Hadropithecus stenognathus *[7,19]. *Hadropithecus *survived until the end of the first millennium A.D. whereas *Archaeolemur *experienced at least a half-a-millennium reprieve [1], possibly thanks to a greater plasticity of resources/habitat exploitation [21]. All of the Archaeolemuridae were extremely powerfully built [19] and exhibited characters reminiscent of cercopithecoids [22]. On the basis of cranial anatomy and dental morphology, Archaeolemuridae have been considered the sister taxon of extant indriids and the palaeopropithecids (families Indriidae and Palaeopropithecidae) [23-25]. However, developmental features [26] as well as the postcranial characters of a newly excavated *Hadropithecus *subadult (Andrahomana Cave, southeastern Madagascar [27,28]) recently challenged this consensual scenario and underscored striking similarities with lemurids (family Lemuridae). The debate is therefore still open [18].

None of the 11 archaeolemurid specimens analysed so far by molecular biologists yielded amplifiable DNA [18]. In this study, we undertook an extensive analysis of 12 new *Archaeolemur *and *Hadropithecus *subfossil remains. We report for the first time the successful characterization of 94–539 bp along two mitochondrial genes (cytochrome *b *and 12S rRNA). This sequence data provides a good support for a sistership between archaeolemurids and extant indriids, in agreement with the most generally accepted morphological phylogenetic scenario but in contrast to recent suggestions made on the basis of analyses of dental developmental and postcranial data.

## Results

To further investigate the evolutionary history of giant lemurs, we performed an ancient DNA study on 12 subfossil remains representing 10 individuals belonging to three extinct giant lemur genera (Table 1). Short, overlapping segments of three mitochondrial genes (control region, cytochrome *b *and 12S rRNA) and one nuclear gene (IRBP) were targeted by PCR (Table S1, Additional file 1). The maximal amplicon length was about 160 bp (including primers). Among the 12 specimens analyzed, only 5 yielded amplifiable DNA although numerous extractions and PCR reactions were performed (Table S2, Additional file 1). It is noteworthy that none of the Control Region fragments targeted gave positive results, suggesting poor efficiency of the PCR primers used here. Even for samples that delivered authentic mtDNA fragments, no nuclear DNA was recovered although fragments as short as 80 bp were targeted (Table S2, Additional file 1). The specimen under collection number 1937–44 at the MNHN yielded authentic DNA fragments when a bone fragment (sample CH147) was sampled but no result when a molar root (sample CH190) was analyzed (Table 1 & Table S2, Additional file 1). All in all, these results suggest poor DNA preservation conditions in the subfossils, which is consistent with what is known about DNA decay in warm subtropical environments [29] and with a previous ancient DNA survey of Malagasy subfossils [18,30].

**Table 1:** Subfossil samples examined.

Species	Sample	**DNA length **(Nb. of overlapping fragments)		Location	Collection Reference	Description
		**Cyt b**	**12S rRNA**			

*Archaeolemur *sp.	CH70	-	-	Antsingiavo-A, Narinda	CNRS UP2147 ref. ATA 2'01	Iliac
	CH71	-	-	Antsingiavo-A, Narinda	CNRS UP2147 ref. ATA 2'01	Iliac
	CH125	-	-	Madagascar	MNHN^2 ^no ref.	Right Femur
*Archaeolemur edwardsi*	CH126	190 (2)	-	Ménagerie	MNHN^2 ^ref. 1931–6	Left Canine sup.
*Archaeolemur majori*	CH127	-	-	Madagascar	MNHN^2 ^ref. MAD57-1906-16	2^nd ^left Molar inf.
	CH145	-	-	Mitoho, Madagascar	MNHN^1 ^ref. 1938–537	Maxilla
	CH146	335 (4)	222 (2)	Madagascar	MNHN^1 ^ref. 1935–419	Molar
	CH191	-	-	Madagascar	MNHN^1 ^ref. 1935–420	Tooth
	CH210	269 (3)	-	unknown	MNHN^1 ^ref. 1936–200	Tooth inf.
*Hadropithecus stenognathus*	CH421	94 (1)	-	Madagascar	MNHN^1 ^ref. 1935–408	Tooth
*Megaladapis edwardsi*	CH147	190 (2)	-	Madagascar	MNHN^1 ^ref. 1937–44	Bone
	CH190	-	-	Madagascar	MNHN^1 ^ref. 1937–44	Molar

MNHN^1 ^= Museum National d'Histoire Naturelle, Bâtiment d'Anatomie Comparée, CP 55 – 55, rue Buffon, 75005 Paris.

MNHN^2 ^= Museum National d'Histoire Naturelle, Dpt. Histoire de la Terre, USM203/UMR5443 Paléobiodiversité, 8 rue Buffon, 75005 Paris.

CNRS UP2147 = Dynamique de l'Évolution Humaine: Individus, Populations, Espèces, 44 rue de l'Amiral Mouchez, 75014 Paris

Column 'Ancient DNA' summarizes the samples that gave authentic ancient DNA fragments.

**Figure 1:**
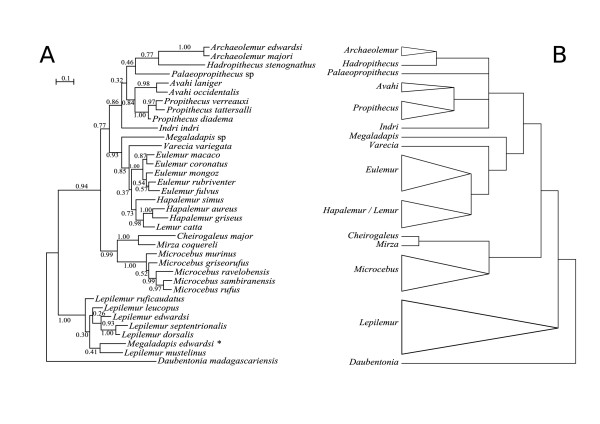
**Phylogenetic relationships among lemuriforms**. **(A) **Phylogenetic tree recovered after Bayesian analysis of Dataset #12 using different model parameters for 12S and Cytb genes. Numbers near the nodes refer to posterior probabilities. * *Megaladapis *haplotype described in Montagnon *et al*. (2001) [31] (Accession number Genbank:AJ278142), criticized in Karanth *et al*. (2005) [18] and definitively confirmed here as a probable PCR-contaminant. **(B) **Summary Consensus of all the phylogenetic trees recovered from the analysis of our 12 datasets.

Despite such difficulties, we managed to recover authentic ancient DNA sequences from individuals belonging to each of the three genera sampled (*Archaeolemur*, *Hadropithecus *and *Megaladapis*). Sample CH147 (*Megaladapis edwardsi*) allowed the recovery of a 190-bp cytochrome *b *sequence that exhibited one and three transitions with the two *Megaladapis *sequences reported in [18] (Accession numbers Genbank:AY894790 and AY894791, respectively). Notably, all these sequences were highly divergent (34 substitutions) from the *Megaladapis *haplotype described in [31] (Figure 1a, noted with a star). This haplotype (Accession number Genbank:AJ278142) was already criticized by Karanth *et al*. (2005) [18] and is now definitively confirmed as a probable PCR-contaminant. *Archaeolemur majori *samples (CH210 and CH146) delivered respectively 269-bp and 335-bp in cytochrome *b *(Table 1). Both sequences were identical over the 269 shared nucleotides but markedly different from all available lemur sequences. *Archaeolemur edwardsi *sample CH126 instead yielded a 190-bp cytochrome *b *sequence (Table 1) that exhibited only a minimum number of 4 substitutions with *Archaeolemur majori *haplotypes while the 94-bp cytochrome *b *sequence obtained from *Hadropithecus stenognathus *sample CH421 was found to exhibit a larger divergence from the *Archaeolemur *sequences (24 substitutions). Raw divergences were in good agreement with what was expected from morphological similarities between these species and genera [19,22]. Because our procedures respect the most stringent criteria of authenticity (independent extractions/amplifications, cloning and sequencing; see Materials and methods), we are confident that the ancient DNA sequences reported here are authentic and we used them in phylogenetic analyses (Figure 1ab).

**Figure 2:**
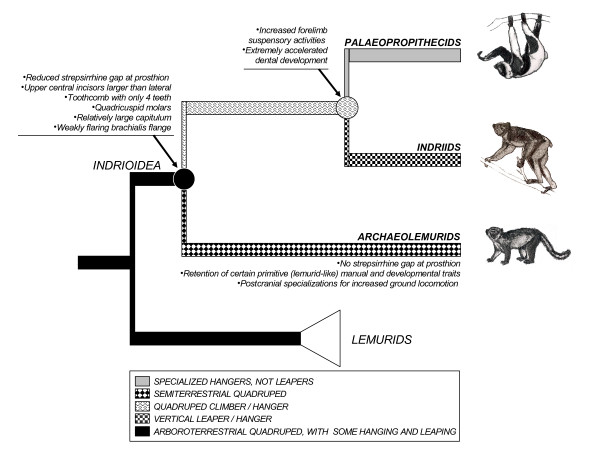
Model for the evolution indrioids, modified from Godfrey (1988).

Accordingly, we retrieved all strepsirrhine cytochrome *b *and 12S rRNA sequences available in Genbank and generated 12 different datasets (Table 2 and Additional data file: for a complete list of the different sequences used). For each of these datasets, phylogenetic trees were built using both maximum likelihood and bayesian methods. Including the new *Archaeolemur *and *Hadropithecus *sequences in the dataset confirmed the indisputable monophyly of Malagasy Lemuriformes. This provided supplemental support to the scenario of a single origin for all Malagasy Primates [32] (reviewed in [33]) and therefore to the authenticity of our sequences (the 'phylogenetic sense' criterion; discussed for instance in [34]). Furthermore, most phylogenies showed the best bootstrap values and posterior probabilities for a sistership between *Archaeolemur *(and *Hadropithecus*) and indriids (up to 86.0% and 0.99, respectively; Table 2, summarized in Figure 1b). Alternative topologies relating *Archaeolemur *(and *Hadropithecus*) to either lemurids, cheirogaleids, lepilemurids or lorisiformes received no more than marginal bootstrap values or posterior probabilities (Table 2). Therefore, regardless of the phylogenetic method or the sequence dataset used, the ancient DNA sequences recovered in this study supported the existence of an Archaeolemuridae-Indriidae clade (Table 2; Figure 1b). Interestingly, despite significant variation among datasets (Table S3, Additional file 1), Approximately Unbiased (AU, [35]) and KH tests [36] come to similar conclusions by rejecting the 3 latter alternative topologies and by showing maximal p-values for the sistership between *Archaeolemur *(and *Hadropithecus*) and indriids. It is noteworthy, also, that the molecular topology presented here (Figure 1b) matches exactly that presented for extant taxa only by DelPero *et al*. (2004) [37] on page 440) as one of the "two remarkably similar topologies that were strongly supported at most of the internal nodes". However, a phylogenomic toolkit using extensive nuclear and mitochondrial sequence data came to different conclusions regarding the position of *Propithecus *(family Indriidae) and *Lepilemur *(family Lepilemuridae) [38]. Interestingly, the former position shows conflict between loci since three of them strongly support the classical view of a sistership of *Propithecus *and lemurids. Furthermore, the authors of this analysis note possible taxonomic bias in their analysis since except for *Propithecus*, no taxa from the family Indriidae (e.g. *Indri *and *Avahi*) have been considered. This added to the limited amount of sequence information used in our study could explain these discrepancies. In any case, the sequences presented in this study sustain the sistership between *Archaeolemur *(and *Hadropithecus*) and indriids as the most likely phylogenetic scenario. Consequently, we can now define a true series of synapomorphies for archaeolemurids, palaeopropithecids, and indriids (collectively, the Indrioidea) at both the dental and postcranial levels (see Figure 2 and Additional data file: "Defining a series of synapomorphies for the Indrioidea clade" for a list of such synapomorphies).

**Table 2:** Sequence datasets and phylogenetic supports for different phylogenetic hypotheses.

Method	Dataset #	Gene	Root	Length	Taxa	indriids	lemurids	cheirogaleids	lepilemurids	lorisiformes	aye-aye
**Likelihood**	1	Cytb	Lorisiformes + Aye-aye	1140	125	21	0	0	0	0	2.5
	2	Cytb	Lorisiformes + Aye-aye	486	125	22.5	0	0.5	0	0	3
	3	Cytb	Aye-aye	486	99	18.5	0.5	0	0	NA	10.5
	4	Cytb	Aye-aye	486	99	0.7	0	0.7	0	NA	4
	5	12S	Lorisiformes + Aye-aye	985	124	72.5	0	3	0	0	1
	6	12S	Lorisiformes + Aye-aye	333	124	46.5	0.5	10.5	0	0	0
	7	12S	Aye-aye	333	89	42	5.5	9	0	NA	1
	8	12S	Aye-aye	197	89	36	4	5	0	NA	2
	9	12S	Aye-aye	372	89	86	0	0.5	0	NA	0.5
	10	12S + Cytb	Lorisiformes + Aye-aye	1934	49	39.5	0	0	0	0	0.5
	11	12S + Cytb	Lorisiformes + Aye-aye	819	49	33.5	0	0	0.5	0	1.5
	12	12S + Cytb	Aye-aye	819	36	33	0	1.5	1.5	NA	2

**Bayesian**	1	Cytb	Lorisiformes + Aye-aye	1140	125	0.29	0	0	0	0	0.22
	2	Cytb	Lorisiformes + Aye-aye	486	125	0.52	0	0	0	0	0
	3	Cytb	Aye-aye	486	99	0.09	0	0	0	NA	0.64*
	4	Cytb	Aye-aye	486	99	0.44	0	0	0	NA	0.26
	5	12S	Lorisiformes + Aye-aye	985	124	0.84	0	0	0	0	0
	6	12S	Lorisiformes + Aye-aye	333	124	0.55	0	0.06	0	0	0
	7	12S	Aye-aye	333	89	0.54	0.06	0.09	0	NA	0
	8	12S	Aye-aye	197	89	0.53	0.08	0	0	NA	0
	9	12S	Aye-aye	372	89	0.99	0	0	0	NA	0
	10	12S + Cytb	Lorisiformes + Aye-aye	1934	49	0.64	0	0	0	0	0
	11	12S + Cytb	Lorisiformes + Aye-aye	819	49	0.68	0	0	0	0	0
	12	12S + Cytb	Aye-aye	819	36	0.88	0	0	0	NA	0

**Bayesian partitioned**	10	12S + Cytb	Lorisiformes + Aye-aye	1934	49	0.63	0	0	0	0	0
	11	12S + Cytb	Lorisiformes + Aye-aye	819	49	0.68	0	0	0	0	0
	12	12S + Cytb	Aye-aye	819	36	0.86	0	0	0	NA	0

Phylogenetic supports for the nesting of *Archaeolemur *(and *Hadropithecus*) within different taxa (Indriidae, Lemuridae, Cheirogaleidae, Lepilemuridae, Lorisiformes or the Aye-aye, respectively) are given in the 6 last columns. Bootstrap percentages or Posterior probabilities are given for Likelihood and Bayesian analyses, respectively. * In this topology, *Archaeolemur *actually appears as the sister taxon of the aye-aye but both are nested within paraphyletic indriids. This finding most likely results from a Long Branch Attraction artifact.

Interestingly, if our molecular data unambiguously link archaeolemurids to indriids, they do not give insights into the phylogenetic relationships within the Indrioidea, as ingroup nodes do not receive conclusive bootstrap values and posterior probabilities (Figure 1A and Table S4, Additional file 1). However, morphological and developmental characters have recently provided unambiguous support for considering the palaeopropithecids as the sister to the Indriidae (*contra *treating the Archaeolemuridae as the sister to the Indriidae) (Figure 2; see Additional data file: "Phylogenetics relationships within Indrioidea" for in-depth discussion). For this reason, this is our preferred phylogeny.

## Discussion

The idea that the Archaeolemuridae, Palaeopropithecidae and Indriidae comprise a clade within the Lemuriformes is not new. Indeed, affinities of *Archaeolemur *to indriines were recognized by Lorenz von Liburnau in 1900 when he named "*Protoindris globiceps*" on the basis of a photograph of a skull (later synonymized with *Archaeolemur majori*) that had been collected by Franz Sikora in 1899 at Andrahomana Cave in southeastern Madagascar [39]. Standing (1908) treated *Archaeolemur *as an indriid [40], and G. E. Smith (1908) discussed the indriid character of its brain [41]. Since that time, craniodental studies have consistently recognized the phylogenetic affinity of Indrioidea and their separation from other lemurs. Often, the three families are treated as subfamilies within the family Indriidae, which in turn is placed within the Lemuroidea. In their review of the craniodental evidence, Tattersall and Schwartz (1974) came to the same conclusion [25]. However, they and other researchers since have recognized that the clade as a whole is not supported by a large number of morphological traits, and that different suites of morphological traits might be used to defend very different phylogenetic hypotheses. Moreover King *et al*. (2001) examined the sequence of fusion of postcranial epiphyses, dental eruption, and closure of cranial sutures in *Archaeolemur *and two living lemurs (*Propithecus *and *Eulemur*) in an effort to evaluate functional and phylogenetic implications of developmental data [26]. These authors noted that the sequence data failed to demonstrate similarities of *Archaeolemur *to *Propithecus*, but rather showed greater likeness to *Eulemur*. In addition, studies of recently-found carpal elements of *Palaeopropithecus*, *Archaeolemur*, and *Hadropithecus *have demonstrated greater likenesses of the archaeolemurids to lemurids [22,27,28,42].

Given that the Archaeolemuridae-Lemuridae sistership is not supported by our molecular data, one interpretation is that the lemurid-likenesses of the Archaeolemuridae are primitive (symplesiomorphic) for indriids, palaeopropithecids, archaeolemurids, and lemurids. Another is that they represent convergences of archaeolemurids and lemurids due to shared quadrupedalism, although this cannot account for developmental likenesses. Godfrey (1988) reconstructed the common ancestor of the Indrioidea as a versatile and probably arboroterrestrial quadruped with limb proportions and a positional repertory not very different from those of the lemurids, *Varecia *or *Pachylemur *[14]. Such a scenario (Figure 2) would explain the manual similarities of the Archaeolemuridae and the Lemuridae. This author also reconstructed the common ancestor of the indriids and palaeopropithecids as a generalized quadrupedal climber/hanger, with striking synapomorphies of the upper limb, hands, and feet. According to this interpretation, an initial split divided the Indrioidea into two clades, one of which (the Archaeolemuridae) specialized in terrestrial quadrupedalism while the other (the palaeopropithecid-indriid clade) specialized in slow quadrupedal climbing and hanging. The latter subsequently split into two clades, one of which (the Palaeopropithecidae) sacrificed rapid locomotion of any sort to perfect deliberate hanging skills, the other of which (the Indriidae) sacrificed quadrupedalism to develop a new form of 'vertical clinging and leaping' while retaining sloth-like hanging skills.

This unique combination of locomotor/postural features and dental adaptations in archaeolemurids is probably the reason why their phylogenetic status has been so difficult to decipher. This study demonstrates the value of the ancient DNA approach in solving the phylogenetic relationships among extinct and extant taxa, especially in situations involving rampant morphological homoplasy, morphological plasticity with rapid change in body size [43,44], or sexual dimorphism [45,46].

## Conclusion

We have been successful in amplifying and sequencing the first ancient DNA sequences of all the members of an enigmatic lemur family: the archaeolemurids (genera *Archaeolemur *and *Hadropithecus*). These 'giant lemurs' lived in Madagascar centuries ago but have been led to extinction by human activities. Our new sequences solve the phylogenetic position of archaeolemurids as close relatives of both the sloth lemurs and the indriids. This appears in sharp contrast with most recent ontogenetic studies as well as new discoveries of postcranial elements of the archaeolemurids that indicate striking similarities with lemurids. In light of our new phylogenetic framework, we were able to reinterpret the available cranial and postcranial data. Our data offer support for a particular scenario of the evolution of the Indrioidea locomotor apparatus (starting from arboroterrestrial ancestors that specialized in either terrestrial quadrupedalism or arboreal skills).

## Methods

### Ancient DNA extraction, amplification and sequencing

A total of 10 samples of subfossil lemurs belonging to the genera *Archaeolemur *and *Hadropithecus *were subjected to ancient DNA extraction (Table 1). These cover all the species currently allied to Archaeolemuridae. Furthermore, two subfossils of the extinct lemur *Megaladapis edwardsi *were also included in the analysis in order to compare the sequences retrieved with the sequences already reported by other laboratories [18,31]. DNA was extracted and amplified as previously described elsewhere [47,48], using appropriate ancient DNA techniques and respective of the most scrupulous ancient DNA authentication criteria [49]. Briefly, mock extractions and the three different amplification controls described in Loreille *et al*. (2001) [50] were included in each analysis to detect possible contamination. Only one lemur sample was extracted per extraction session to limit possible cross-contamination between specimens. All PCR reactions were conducted in a total volume of 25–100 μl using 2.5–10 units of Taq Gold (Perkin-Elmer^®^) together with 2 mM MgCl2, 1 mg/ml BSA, 250 μM of each dNTP and 0,5–1 μM of the different primers listed in Table S1, Additional file 1. A 5–10 min activation step at 92–94°C was followed by 50–60 cycles of denaturation (92–94°C, 45–60s), annealing (44–50°C, 45–60s), extension (72°C, 45s) and a last extension step at 72°C (5–10 min). Primers (Table S1, Additional file 1) were designed to target overlapping DNA fragments of 80–200 bp among 3 different mtDNA genes (control region, cytochrome *b *and 12S rRNA) and one nuclear gene (IRBP). No specimen or DNA extract from modern lemurs was ever introduced in the laboratories. PCR products were cloned using the Topo TA cloning kit (Invitrogen^®^) following the manufacturer instructions. Colonies positive for insertion were screened by PCR into a 12 μl reaction mix using universal M13 (5'-GTT TTC CCA GTC ACG ACG TTG) and REV (5'-TTT CAC ACA GGA AAC AGC TAT) primers and 35–45 cycles of denaturation (94°C, 30s), annealing (55°C, 30s) and elongation (72°C, 45s). PCR products were further sequenced by a service provider (Cogenics^®^). For each DNA fragment, the final sequence was deduced from the consensus of all clone sequences obtained from at least two independent PCR products. Such an approach is generally taken for discarding possible artifactual substitutions induced by DNA damage [51]. A total number of 75 PCR products and 399 clones were analyzed (Table S2, Additional file 1). For cytochrome *b*, no prematurate stop-codon is observed in the coding-phase of each of the final consensus. Finally, the sample CH146 (*Archaeolemur majori*) was independently extracted, amplified and analysed in two different ancient DNA laboratories. It yielded identical consensus cytochrome *b *sequences.

### Datasets

The new sequences reported in this manuscript were deposited in Genbank under Accession numbers EU441938–EU441943. All available sequences of extinct and extant Strepsirrhini were retrieved from Genbank and aligned using ClustalW. In order to investigate possible artifacts due to stochastic or systematic errors, 12 different datasets were constituted. Dataset composition is provided in the Additional data file.

### Phylogenetic analyses

Bayesian Markov Chain Monte Carlo phylogenies were generated using MrBayes 3.12 [52] under a GTR model of evolution assuming a fraction of invariant sites and rate heterogeneity across sites. Two sets of four chains sampled every 100 generations were run until the average standard deviation of split frequencies between the two sets fell below the default critical value of 0.01 using a burn-in fraction of 25%. Bayesian posterior probabilities were finally recorded even if their significance, in term of robustness, remains an open question (e.g. [53]). For each dataset, the best-fitting model of substitution was then determined using Modeltest [54] following AIC criterion [55]. Maximum Likelihood (ML) trees were then built with Phyml [56]. The strength of the phylogenetic signal was assessed via non-parametric bootstrapping [57] among 200 pseudo replicates. For datasets #10–12, we analyzed either both genes under the same (Likelihood and Bayesian, respectively) or independent (Bayesian partitioned) model parameters (Tables 2 & S4, Additional file 1). Statistical supports for different *a priori *selected hypotheses were assessed via the Approximately Unbiased test (AU, [35]) and unilateral KH test [36] using Consel [58].

## Authors' contributions

LO, SC and CS extracted, amplified and sequenced ancient DNA. LG provided palaeontological information. CD performed the phylogenetic analyses. CH initiated and coordinated the study. LO and LG wrote the paper.

## Supplementary Material

Additional file 1 **Table S1**. Fragment length, primer sequences and T annealing of the PCR fragments targeted under this study.
**Table S2.** Number of independent PCR amplifications and clones sequenced per fragment and per sample.
**Table S3.** p-values of AU and KH tests for the clustering of *Archaeolemur* (and *Hadropithecus*) within different taxa (indriids, lemurids, cheirogaleids, lepilemurids, lorisiformes or aye-aye, respectively) or outside indriids (no indriids).
**Table S4.** Phylogenetic supports for three alternative relationships among Indrioidea. Click here for file
