# Supplementary material for: DNA from extinct giant lemurs links archaeolemurids to extant indriids
Source: BMC Evol Biol. 2008 Apr 28;8:121. doi: 10.1186/1471-2148-8-121 (PMC2386821; doi:10.1186/1471-2148-8-121)
Supplement: Additional file 1 — Table S1. Fragment length, primer sequences and T annealing of the PCR fragments targeted under this study. Table S2. Number of independent PCR amplifications and clones sequenced per fragment and per sample. Table S3. p-values of AU and KH tests for the clustering of Archaeolemur (and Hadropithecus) within different taxa (indriids, lemurids, cheirogaleids, lepilemurids, lorisiformes or aye-aye, respectively) or outside indriids (no indriids). Table S4. Phylogenetic supports for three alternative relationships among Indrioidea. [file 1471-2148-8-121-S1.doc]

**Additional data file**

| **Gene** | **Primer 1 (5’-3’)** | | **Primer 2 (5’-3’)** | | **Length (bp)^$^** |
| --- | --- | --- | --- | --- | --- |
|  | **Sequence** | **Position** | **Sequence** | **Position** |  |
|  |  |  |  |  |  |
| **Cytochrome b** | ATG AMY AAC ATC CGA AAR A | 14176-14194 | ATT TGT AGG RYT AGG CAG GCT | 14289-14309 | 134 |
| * | *TCT AAT ATC TCC TCA TGA TGA A* | 14248-14269 | *TCA GCC GTA GTT TAC RTC WCG GCA* | 14383-14406 | 159 |
| * | tag caa tac act aca cag cag | 14327-14347 | TCG GCC TAC ATG GAT GAA TA | 14456-14475 | 149 |
| * | GCC AAC GGA GCA TCC ATA TT | 14425-14444 | AAG GCT GTG GCT ATT ACT GT | 14539-14558 | 134 |
| * | TAC TAT GGC TCC TTC ATA TTC TCA A | 14482-14506 | AGR TTT GTR ATY ACT GT | 14605-14621 | 140 |
|  | *GAA CAC CAA TGA MCA AYA TYC GA* | 14168-14190 | *AKR GCT ARG CAG GCY CCT AGT AGG* | 14280-14303 | 136 |
|  | *GAA CAC CAA TGA MCA AYA TYC GA* | 14168-14190 | AGG ATT AGG CAG GTC CCT AGA AGG | 14280-14303 | 136 |
|  | *CAT TAT CAG AAA CCT GAA ATA TTG G* | 14498-14522 | *AAT GTA TGG GAT TGC TGA GAG TAG G* | 14619-14643 | 146 |
|  | TAT TCT CAA AAA CTT GAA ACA CAG G | 14498-14522 | AGR TTK GTR ATT ACT GT | 14605-14621 | 124 |
|  |  |  |  |  |  |
| **12S rRNA** | GAT TAG ATA CCC RAR TAT GCT T | 491-512 | TTT ATC GGG GTT TAT CGA TTA T | 627-648 | 158 |
| * | CTA TAA ATC GAT AAA CCC CGA TA | 625-646 | TTC TTY CCA CTT CAT AGA | 759-776 | 153 |
| * | GGT GTA GTC TAT GAA GTG G | 752-770 | TCA ATT AAG CTC TCT ATT CTT | 864-884 | 133 |
|  |  |  |  |  |  |
| **Control Region** | CAC CTT CAR CMC CCA AAG CTG | 15409-15429 | gga arg gcr cak nat gca cga | 15487-15507 | 99^£^ |
|  | TCG TAC ATA CAN YTC YAG YC | 15566-15583 | NTG TNC GAT YAA TKK AGR RAT | 15710-15730 | 165^£^ |
|  | TCG TAC ATA CAI YTC YAG YC | 15566-15583 | NTG TNC GAT YAA TKK AGR RAT | 15710-15730 | 165^£^ |
|  | ACG CAT ATA AGC CAG TAC AT | 15589-15608 | GAC TTT GAA ATA GGA TGC GC | Not aligned | 117^£^ |
|  | GGC TAT CCA CAA CCT AAT CAA TCG | 15589-15608 | GAC TTT GAA ATA GGA TGC GC | Not aligned | 117^£^ |
|  | GCA TAT AAG CYA GTA CAT WA | 15669-15688 | GAA ATA GGA TGC GCT AAT GTG | 15726-15746 | 77^£^ |
|  | CCA CAA CCT AAT CAA TCG C | Not aligned | GAA ATA GGA TGC GCT AAT GTG | 15726-15746 | 105^£^ |
|  | AYA TRR NAC ATY YCY MMA TTR ATC G | 15701-15725 | STG GTT TCA CGG AGG ATG GTAG | 15797-15818 | 118^£^ |
|  | AYA TRR IAC ATY YCY MMA TTR ATC G | 15701-15725 | TGR TTT CAC GGA GGA TGG TAG | 15797-15817 | 117^£^ |
|  |  |  |  |  |  |
| **IRBP** | GAG GAC CCC AGG CTC CTG GTG | 1018-1038 | TCG TCC TCG GGC ACC TCT GRG | 1107-1127 | 110 |
|  | RGR CCC AGR GAA ACC CYT CC | 1048-1067 | TCG TCC TCG GGC ACC TCT GRG | 1107-1127 | 80 |

**Table S1.** PCR amplifications: fragment length and primer sequences.

Primers described in Karanth et al. (2005) are indicated in italics. Primer positions and amplicon lengths are given with respect to the *Lemur catta* reference sequences AJ421451 for mitochondrial genes and AJ313470 for IRBP gene. *Delivered authentic aDNA fragments. Grey shaded: delivered no PCR products or artifactual PCR by-products only. ^£^When the fragment targeted may include some indels, the maximal length expected is given. N = A,C,G or T. Y = C or T. R = A or G. S = C or G. W = A or T. M = A or C.V = A,C or G. K = G or T. I stands for Inosine. ^$^ Primers included (the length is given with reference to the complete mtDNA genome of the ring-tailed lemur). Not aligned: primers exhibiting sequence similarity with other lemurs (such as *Propithecus tattersalli*) but not with *Lemur catta*.

|  |  |  | **Operator (A / C)*** | | |
| --- | --- | --- | --- | --- | --- |
| **Primer forward** | **Primer reverse** | **Sample** | **CS, LAB#1** | **LO, LAB#2** | **SC, LAB#2** |
|  |  |  |  |  |  |
| **Cytochrome b** |  |  |  |  |  |
|  |  |  |  |  |  |
| 14248-14269 | 14383-14406 | CH146 | n/a | 6 / 48 | n/a |
|  |  | CH210 | n/a | n/a | 2 / 12 |
|  |  |  |  |  |  |
| 14327-14347 | 14456-14475 | CH126 | 10 / 29 | - | - |
|  |  | CH146 | 6 / 12 | 3 /28 | n/a |
|  |  | CH147 | 1 / 5 | - | - |
|  |  | CH210 | n/a | n/a | 2 / 14 |
|  |  |  |  |  |  |
| 14425-14444 | 14539-14558 | CH126 | 14/ 50 | - | - |
|  |  | CH146 | 6 / 12 | 3 / 20 | n/a |
|  |  | CH147 | 5 / 14 | - | - |
|  |  | CH210 | n/a | n/a | 2 / 14 |
|  |  | CH421 | n/a | 2 / 17 | 3 / 24 |
|  |  |  |  |  |  |
| 14482-14506 | 14605-14621 | CH146 | n/a | 2 / 23 | n/a |
|  |  |  |  |  |  |
| **12S rRNA** |  |  |  |  |  |
|  |  |  |  |  |  |
| 625-646 | 759-776 | CH146 | n/a | 2 / 11 | n/a |
|  |  |  |  |  |  |
| 752-770 | 864-884 | CH146 | n/a | 4 / 42 | 2 / 24 |

**Table S2.** Number of independent PCR amplifications and clones sequenced per fragment and per sample.

The experiments were conducted in two different ancient DNA laboratories: one located at the University Claude Bernard (UCB Lyon1, LAB#1) and the other at the Ecole Normale Supérieure de Lyon (ENS de Lyon, LAB#2). Three different operators were involved in the experiments. * (A / C), Above : Number of independent amplifications (A), Below: number of clones (C) sequenced. n/a not attempted. -: attempted but negative results.

| **Test** | **Dataset #** | **Gene** | **Root** | **Length (nt)** | **Taxa** | **indriids** | **no indrids** | **lemurids** | **cheirogaleids** | **lepilemurids** | **lorisiformes** | **aye-aye** |
| --- | --- | --- | --- | --- | --- | --- | --- | --- | --- | --- | --- | --- |
| AU | 1 | Cytb | Lorisiformes + Aye-aye | 1140 | 125 | 0.616 | 0.234 | 0.174 | 0.059 | 0.054 | 0.060 | 0.141 |
|  | 2 | Cytb | Lorisiformes + Aye-aye | 486 | 125 | 0.566 | 0.634 | 0.116 | 0.077 | 0.062 | 0.064 | 0.557 |
|  | 3 | Cytb | Aye-aye | 486 | 99 | 0.662 | 0.505 | 0.150 | 0.075 | 0.044 | NA | 0.500 |
|  | 4 | Cytb | Aye-aye | 486 | 99 | 0.635 | 0.576 | 0.132 | 0.016 | 0.051 | NA | 0.458 |
|  | 5 | 12S | Lorisiformes + Aye-aye | 985 | 124 | 0.605 | 0.614 | 0.342 | 0.120 | 0.011 | 0.300 | 0.011 |
|  | 6 | 12S | Lorisiformes + Aye-aye | 333 | 124 | 0.725 | 0.223 | 0.569 | 0.594 | 0.002 | 0.197 | 0.063 |
|  | 7 | 12S | Aye-aye | 333 | 89 | 0.637 | 0.556 | 0.396 | 0.372 | 0.002 | NA | 0.002 |
|  | 8 | 12S | Aye-aye | 197 | 89 | 0.582 | 0.548 | 0.426 | 0.353 | 0.002 | NA | 0.005 |
|  | 9 | 12S | Aye-aye | 372 | 89 | 0.842 | 0.225 | 0.339 | 0.200 | 0.004 | NA | 0.004 |
|  | 10 | 12S + Cytb | Lorisiformes + Aye-aye | 1934 | 49 | 0.637 | 0.578 | 0.178 | 0.347 | 0.466 | 0.014 | 0.243 |
|  | 11 | 12S + Cytb | Lorisiformes + Aye-aye | 819 | 49 | 0.762 | 0.558 | 0.096 | 0.074 | 0.041 | 0.025 | 0.039 |
|  | 12 | 12S + Cytb | Aye-aye | 819 | 36 | 0.864 | 0.422 | 0.132 | 0.256 | 0.003 | NA | 0.074 |
| KH | 1 | Cytb | Lorisiformes + Aye-aye | 1140 | 125 | 0.447 | 0.383 | 0.117 | 0.079 | 0.066 | 0.106 | 0.109 |
|  | 2 | Cytb | Lorisiformes + Aye-aye | 486 | 125 | 0.439 | 0.548 | 0.127 | 0.107 | 0.121 | 0.120 | 0.452 |
|  | 3 | Cytb | Aye-aye | 486 | 99 | 0.573 | 0.427 | 0.143 | 0.056 | 0.058 | NA | 0.430 |
|  | 4 | Cytb | Aye-aye | 486 | 99 | 0.562 | 0.438 | 0.134 | 0.022 | 0.057 | NA | 0.389 |
|  | 5 | 12S | Lorisiformes + Aye-aye | 985 | 124 | 0.507 | 0.493 | 0.300 | 0.493 | 0.233 | 0.416 | 0.025 |
|  | 6 | 12S | Lorisiformes + Aye-aye | 333 | 124 | 0.636 | 0.234 | 0.449 | 0.364 | 0.019 | 0.155 | 0.065 |
|  | 7 | 12S | Aye-aye | 333 | 89 | - | 0.400 | 0.395 | 0.361 | 0.012 | NA | 0.012 |
|  | 8 | 12S | Aye-aye | 197 | 89 | 0.531 | 0.399 | 0.381 | 0.326 | 0.011 | NA | 0.014 |
|  | 9 | 12S | Aye-aye | 372 | 89 | 0.829 | 0.186 | 0.171 | 0.160 | 0.011 | NA | 0.011 |
|  | 10 | 12S + Cytb | Lorisiformes + Aye-aye | 1934 | 49 | 0.527 | 0.473 | 0.189 | 0.279 | 0.366 | 0.022 | 0.270 |
|  | 11 | 12S + Cytb | Lorisiformes + Aye-aye | 819 | 49 | 0.579 | 0.421 | 0.078 | 0.061 | 0.030 | 0.025 | 0.045 |
|  | 12 | 12S + Cytb | Aye-aye | 819 | 36 | 0.703 | 0.297 | 0.095 | 0.179 | 0.008 | NA | 0.058 |

**Table S3.** p-values of AU and KH tests for the clustering of *Archaeolemur* (and *Hadropithecus*) within different taxa (indriids, lemurids, cheirogaleids, lepilemurids, lorisiformes or aye-aye, respectively) or outside indriids (no indriids).

| **Method** | **Dataset #** | **Gene** | **Root** | **Length (nt)** | **Taxa** | **Topology A = (I,P)** | **Topology B = (I,(A,H))** | **Topology C = (P,(A,H))** |
| --- | --- | --- | --- | --- | --- | --- | --- | --- |
| **Likelihood** | 1 | Cytb | Lorisiformes + Aye-aye | 1140 | 125 | 11 | 11 | 4 |
|  | 2 | Cytb | Lorisiformes + Aye-aye | 486 | 125 | 7 | 7.5 | 7 |
|  | 3 | Cytb | Aye-aye | 486 | 99 | 6 | 10.5 | 5 |
|  | 4 | Cytb | Aye-aye | 486 | 99 | 0 | 0 | 1 |
|  | 10 | 12S + Cytb | Lorisiformes + Aye-aye | 1934 | 49 | 13.5 | 7.5 | 3.5 |
|  | 11 | 12S + Cytb | Lorisiformes + Aye-aye | 819 | 49 | 7.5 | 3.5 | 9.5 |
|  | 12 | 12S + Cytb | Aye-aye | 819 | 36 | 3 | 8 | 8.5 |
| **Bayesian** | 1 | Cytb | Lorisiformes + Aye-aye | 1140 | 125 | 0 | 0 | 0.36 |
|  | 2 | Cytb | Lorisiformes + Aye-aye | 486 | 125 | 0 | 0 | 0.34 |
|  | 3 | Cytb | Aye-aye | 486 | 99 | 0.05 | 0 | 0.26 |
|  | 4 | Cytb | Aye-aye | 486 | 99 | 0 | 0 | 0.13 |
|  | 10 | 12S + Cytb | Lorisiformes + Aye-aye | 1934 | 49 | 0.06 | 0 | 0.20 |
|  | 11 | 12S + Cytb | Lorisiformes + Aye-aye | 819 | 49 | 0.07 | 0.06 | 0.23 |
|  | 12 | 12S + Cytb | Aye-aye | 819 | 36 | 0.09 | 0.08 | 0.39 |
| **Bayesian** **Partioned** | 10 | 12S + Cytb | Lorisiformes + Aye-aye | 1934 | 49 | 0.09 | 0 | 0.29 |
|  | 11 | 12S + Cytb | Lorisiformes + Aye-aye | 819 | 49 | 0.07 | 0.05 | 0.24 |
|  | 12 | 12S + Cytb | Aye-aye | 819 | 36 | 0.09 | 0.07 | 0.45 |

**Table S4.** Phylogenetic supports for three alternative relationships among Indrioidea.

I: Indriids. P: Paleopropithecids. A: *Archaeolemur*. H: *Hadropithecus*. Bootstrap percentages or Posterior probabilities are given for Likelihood and Bayesian analyses, respectively.

**Defining a series of synapomorphies for the Indrioidea clade (modified from Godfrey, 1988)**

A few dental / postcranial synapomorphies support an indrioid clade:

- the central upper incisors are more closely approximated in members of this group than is typical for other lemurs (i.e., there is less of a strepsirrhine gap at prosthion, and none in the case of the Archaeolemuridae)
- the upper central incisors are typically larger than lateral upper incisors. The adult dentition lacks a lower canine, so that the toothcomb (or modified toothcomb) comprises only four (rather than six) teeth
- the molars are quadricuspid (roughly square), with mesial and distal pairs (protocone, paracone, metacone, and hypocone in the uppers, and protoconid, metaconid, hypoconid and entoconid in the lowers) more or less transversely aligned
- a relatively large capitulum and weakly flaring brachialis flange.

**Phylogenetic relationships within Indrioidea**

During the 1970s and 1980s, Tattersall waivered in his interpretation of the phylogenetic relationships of the three indrioid families (or subfamilies), alternatively treating the palaeopropithecids as the sister to the Indriidae (topology A in Table S4; Tattersall and Schwartz, 1974; Schwartz and Tattersall, 1985), the Archaeolemuridae as the sister to the Indriidae (topology B in Table S4; Tattersall, 1982), or the problem as effectively unresolved (Tattersall, 1973). Unequivocal sister taxon relationships within this group seemed apparent only for *Archaeolemur* and *Hadropithecus*, and for *Palaeopropithecus* and *Archaeoindris* (Standing, 1908; Lamberton, 1929, 1934; Godfrey 1988; Godfrey and Jungers, 2002).

But based on the morphology of the humerus and femur of extinct and extant lemurs (i.e. features that had not been considered in previous analyses), Godfrey (1988) found support for topology A. Despite striking differences in long bone proportions and positional specializations (the Palaeopropithecidae for quadrupedal suspension and the Indriidae for leaping), the Palaeopropithecidae and the Indriidae display a far more significant number of apparent postcranial synapomorphies, which Godfrey (1988) related to a common tendency to employ the forelimb in suspensory activities. Thus, for example, the humeral tubercles are situated below the level of the head of the humerus; the infraspinatus pit is directed cranially, and therefore visible in proximal view; the lesser tubercle projects medially; the humeral head is strongly curved along its sagittal axis; the medial epicondyle is strong, medially projecting and proximally extensive; the trochlea is small and square; the groove separating the trochlea and capitulum is wide; the olecranon fossa is narrow and fairly shallow. An enlarged femoral head articulates with a shallow acetabulum, facilitating thigh rotation at the hip joint.

Recent discoveries of new species of palaeopropithecids (*Babakotia radofilai* and *Mesopropithecus dolichobrachion*), and of previously unknown postcranial elements of species already described, have provided additional evidence in support of sistership for the palaeopropithecids and indriids, to the exclusion of the archaeolemurids (e.g., Godfrey et al., 1990; Jungers et al., 1991, Simons et al., 1995). Yet more support for topology A has emerged in recent years from microstructural studies of dental development of the extinct lemurs (Schwartz et al., 2002; Godfrey et al., 2006ab). Not merely do the Palaeopropithecidae and Indriidae share details of their dental morphology (Tattersall and Schwartz, 1974), but they share extremely accelerated dental development, including very early prenatal initiation of the first molar crown, early completion of the first molar crown, and relatively early replacement of the deciduous molars, in addition to a diminution of the size of the deciduous molars, and the loss of a pair of premolars in the adult dentition (Godfrey et al., 2002; Schwartz et al., 2002, Godfrey et al., 2006b).

**Additional References**

Godfrey LR: **Adaptive diversification of Malagasy strepsirrhines.** *J Hum Evol* 1988 **17**: 93-134.

Tattersall I, Schwartz JH: **Craniodental morphology and the systematics of the Malagasy lemurs (Primates, Prosimii).** *Anthropol Pap Am Mus Nat Hist* 1974 **52**: 139-192.

Schwartz JH, Tattersall I: **Evolutionary relationships of living lemurs and lorises (Mammalia, Primates) and their potential affinities with European Eocene Adapidae.** *Anthrop Pap Am Mus Nat Hist* 1985 **60**: 1-100.

Tattersall I: **The Primates of Madagascar**. New-York: Columbia University Press. 1982 382p.

Tattersall I: **Cranial anatomy of the Archaeolemurinae (Lemuroidea, Primates).** *Anthropol Pap Am Mus Nat Hist* 1973 **52**: 1-110.

#### Standing HF: On recently discovered subfossil Primates from Madagascar. *Trans Zool Soc* 1908 18:163-177.

Lamberton C: **Sur les *Archaeoindris* de Madagascar.** *C R Hebd Séanc Acad Sci Paris* 1929 **188**: 1572-1574.

Lamberton C : **Contribution à la connaissance de la faune subfossile de Madagascar: lémuriens et ratites. L’*Archaeoindris fontoynonti.*** *Stand Mém Acad Malgache (nouv sér)* 1934 **17**: 9-39.

Godfrey LR, Jungers WL: **Quaternary fossil lemurs.** In: Hartwig W, editor. The Primate Fossil Record. New York: Cambridge University Press. 2002 pp. 97-122 (plus end-book references).

Godfrey L, Sutherland MR, Petto AJ, Boy DS: **Size, space, and adaptation in some subfossil lemurs from Madagascar.** *Am J Phys Anthropol* 1990 **81**: 45-66.

#### Jungers WL, Godfrey LR, Simons EL, Chatrath PS, Rakotosamimana B: Phylogenetic and functional affinities of *Babakotia* (primates), a fossil lemur from northern Madagascar. *Proc Natl Acad Sci USA* 1991 88: 9082-9086.

Simons EL, Godfrey LR, Jungers WL, Chatrath PS, Ravaoarisoa J: **A new species of *Mesopropithecus* (Primates, Palaeopropithecidae) from Northern Madagascar.** *Int J Primatol* 1995 **16**: 653-682.

#### Schwartz GT, Samonds KE, Godfrey LR, Jungers WL, Simons EL: Dental microstructure and life history in subfossil Malagasy lemurs. *Proc Natl Acad Sci USA* 2002 99: 6124-6129.

Godfrey LR, Jungers WL, Burney DA, Vasey N, Ramilisonina , Wheeler W, Lemelin P, Shapiro LJ, Schwartz GT, King SJ, Ramarolahy MF, Raharivony LL, Randria GF: **New discoveries of skeletal elements of *Hadropithecus stenognathus* from Andrahomana cave, souteastern Madagascar.** *J Hum Evol* 2006a **51**: 395-410.

Godfrey LR, Schwartz GT, Samonds KE, Jungers WL, Catlett KK: **The secrets of lemur teeth.** *Evolutionary Anthropol* 2006b **15**:142-154.

Godfrey LR, Petto AJ, Sutherland MR: **Dental ontogeny and life history strategies: The case of the giant extinct indroids of Madagascar.** In: Plavcan JM, Kay RF, Jungers WL, van Schaik CP, editors. Reconstructing Behavior in the Primate Fossil Record. New York: Kluwer Academic/Plenum Publishers. 2002 Pp 113-157.

**Datasets. List of the 12 datasets used for phylogenetic inference.**

**Dataset 1: full length (1140 bp) Cytochrome B with 125 Strepsirrhini.**

| Allocebus trichotis | AY441461 |
| --- | --- |
| Archaeolemur edwarsi CH126 (190bp) | EU441939 |
| Archaeolemur majori CH146 (335bp) | EU441940 |
| Arctocebus calabarensis | AY441474 |
| Avahi laniger | AY441453 |
| Avahi occidentalis | AY441454 |
| Cheirogaleus crossleyi | AY605927 |
| Cheirogaleus major | AY605911 |
| Cheirogaleus major | AY605921 |
| Cheirogaleus major | AY441457 |
| Cheirogaleus medius | AY441458 |
| Cheirogaleus medius | AY605903 |
| Daubentonia madagascariensis | AY441444 |
| Daubentonia madagascariensis | U53569 |
| Eulemur coronatus | AY441448 |
| Eulemur fulvus albifrons | AF081048 |
| Eulemur fulvus albocollaris | AF175857 |
| Eulemur fulvus albocollaris | AF175858 |
| Eulemur fulvus collaris | U53576 |
| Eulemur fulvus fulvus | AF175841 |
| Eulemur fulvus fulvus | AF175842 |
| Eulemur fulvus rufus | U53577 |
| Eulemur fulvus sanfordi | AF175846 |
| Eulemur fulvus sanfordi | AF175845 |
| Eulemur macaco flavifrons | AF081050 |
| Eulemur macaco macaco | AF081049 |
| Eulemur mongoz | AY441449 |
| Eulemur mongoz | AF081051 |
| Eulemur rubriventer | AF081052 |
| Euoticus elegantulus | AY441469 |
| Galago alleni | Z35095 |
| Galago gabonensis | AY441467 |
| Galago gallarum | AF212970 |
| Galago granti | AY441468 |
| Galago matschiei | AF271409 |
| Galago moholi | AY441470 |
| Galago moholi | AF271410 |
| Galago senegalensis | AY441471 |
| Galagoides demidoff | AF271411 |
| Galagoides demidoff | AY441472 |
| Galagoides zanzibaricus | AF212964 |
| Hadropithecus stenognathus CH421 (94bp) | EU441943 |
| Hapalemur aureus | AY441446 |
| Hapalemur griseus | U53574 |
| Hapalemur griseus meridionalis | AY441447 |
| Hapalemur simus | AJ428978 |
| Hapalemur simus | AJ428979 |
| Indri indri | AY441455 |
| Lemur catta | AF175960 |
| Lemur catta | AF175959 |
| Lepilemur aeeclis | DQ234899 |
| Lepilemur aeeclis | DQ108999 |
| Lepilemur ankaranensis | DQ109024 |
| Lepilemur ankaranensis | DQ109025 |
| Lepilemur dorsalis | DQ234886 |
| Lepilemur dorsalis | AY441464 |
| Lepilemur edwardsi | DQ109004 |
| Lepilemur edwardsi | DQ109005 |
| Lepilemur leucopus | DQ109007 |
| Lepilemur microdon | DQ109010 |
| Lepilemur microdon | DQ109009 |
| Lepilemur mustelinus | DQ109033 |
| Lepilemur mustelinus | DQ109034 |
| Lepilemur randrianasoli | DQ234894 |
| Lepilemur randrianasoli | DQ234890 |
| Lepilemur ruficaudatus | DQ109017 |
| Lepilemur ruficaudatus | DQ109012 |
| Lepilemur sahamalazensis | DQ108991 |
| Lepilemur sahamalazensis | DQ108992 |
| Lepilemur septentrionalis | DQ109021 |
| Lepilemur septentrionalis | DQ234900 |
| Loris tardigradus | U53581 |
| Loris tardigradus | AY441475 |
| Megaladapis edwarsi CH147 (190bp) | EU441942 |
| Megaladapis sp | AY894795 |
| Megaladapis sp | AY894796 |
| Megaladapis sp | AJ278142 |
| Megaladapis sp | AY894790 |
| Megaladapis sp | AY894791 |
| Microcebus berthae | AY441459 |
| Microcebus berthae | AF285540 |
| Microcebus griseorufus | AY167075 |
| Microcebus griseorufus | AF285567 |
| Microcebus lehilahytsara | DQ095783 |
| Microcebus lehilahytsara | DQ095782 |
| Microcebus murinus | AF285557 |
| Microcebus murinus | AF285565 |
| Microcebus myoxinus | AF285536 |
| Microcebus myoxinus | AF285539 |
| Microcebus ravelobensis | AF285531 |
| Microcebus ravelobensis | AF285529 |
| Microcebus rufus | AF285551 |
| Microcebus rufus | AF285549 |
| Microcebus rufus | AF285553 |
| Microcebus rufus | AF285552 |
| Microcebus sambiranensis | AF285556 |
| Microcebus sambiranensis | AF285554 |
| Microcebus tavaratra | AF285534 |
| Microcebus tavaratra | AF285533 |
| Mirza coquereli | DQ093178 |
| Mirza coquereli | DQ093175 |
| Mirza zaza | DQ093169 |
| Mirza zaza | DQ093170 |
| Nycticebus bengalensis | AY441477 |
| Nycticebus coucang | NC002765 |
| Nycticebus coucang | U53580 |
| Nycticebus pygmaeus | AY687900 |
| Nycticebus pygmaeus | AY441476 |
| Otolemur crassicaudatus | U53579 |
| Otolemur crassicaudatus | AY441465 |
| Otolemur garnettii | AY441466 |
| Otolemur garnettii | AF271412 |
| Palaeopropithecus sp | AY894794 |
| Palaeopropithecus sp | AY894792 |
| Palaeopropithecus sp | AY894793 |
| Perodicticus potto | AY441473 |
| Perodicticus potto | AF271413 |
| Phaner furcifer | AY441456 |
| Propithecus diadema diadema | AY441452 |
| Propithecus tattersalli | U53573 |
| Propithecus verreauxi | AF285528 |
| Propithecus verreauxi | AY441451 |
| Varecia variegata rubra | AY441450 |
| Varecia variegata rubra | U53578 |
| Varecia variegata variegata | AF081047 |

**Dataset 2: as in Dataset 1 but for a 486 bp subset of the Cytochrome B.**

This data minimizes the amount of missing data.

**Dataset 3: as in Dataset 2 but for a subset of the Lemuriformes.**

Lorisiformes were excluded to limit saturation effects and systematic errors associated. The tree was rooted on *Daubentonia madagascariensis*.

**Dataset 4: as in Dataset 3 but third cordon position were RY coded in order to reduce saturation artifact.**

**Dataset 5: full length (985 bp) 12S rRNA with 124 Strepsirrhini.**

| Archaeolemur majori CH146 (222bp) | EU441938 |
| --- | --- |
| Arctocebus aureus | DQ073478 |
| Arctocebus aureus | DQ073477 |
| Arctocebus calabarensis | DQ073479 |
| Avahi laniger | AJ429616 |
| Avahi occidentalis | AJ429617 |
| Avahi occidentalis | AY043343 |
| Cheirogaleus major | AJ429627 |
| Cheirogaleus major | AY043347 |
| Daubentonia madagascariensis | AY043348 |
| Daubentonia madagascariensis | AF175781 |
| Eulemur coronatus | AY043335 |
| Eulemur coronatus | AF175773 |
| Eulemur coronatus | AF175772 |
| Eulemur fulvus | DQ073499 |
| Eulemur fulvus albifrons | AF175797 |
| Eulemur fulvus albocollaris | AF175799 |
| Eulemur fulvus albocollaris | AF175798 |
| Eulemur fulvus collaris | AF175775 |
| Eulemur fulvus collaris | AF175774 |
| Eulemur fulvus fulvus | AF175783 |
| Eulemur fulvus fulvus | AF175782 |
| Eulemur fulvus rufus | AF175795 |
| Eulemur fulvus rufus | AF175796 |
| Eulemur fulvus sanfordi | AF175786 |
| Eulemur fulvus sanfordi | AF175787 |
| Eulemur macaco | AY043336 |
| Eulemur macaco flavifrons | AF175777 |
| Eulemur macaco flavifrons | AF175776 |
| Eulemur macaco macaco | AF175790 |
| Eulemur macaco macaco | AF175778 |
| Eulemur mongoz | AY043338 |
| Eulemur mongoz | AF175785 |
| Eulemur mongoz | AF175784 |
| Eulemur rubriventer | AF175788 |
| Eulemur rubriventer | AY043337 |
| Eulemur rubriventer | AF175789 |
| Euoticus elegantulus | DQ073480 |
| Euoticus legantulus | AY897396 |
| Galago | AF212950 |
| Galago alleni | DQ073523 |
| Galago alleni | DQ073522 |
| Galago alleni | DQ073521 |
| Galago moholi | DQ073481 |
| Galagoides demidoff | DQ073494 |
| Galagoides demidoff | DQ073493 |
| Galagoides demidoff | DQ073489 |
| Galagoides granti | DQ073520 |
| Galagoides orinus | DQ073498 |
| Galagoides zanzibaricus | DQ073497 |
| Galagoides zanzibaricus | DQ073496 |
| Hapalemur aureus | AJ430036 |
| Hapalemur aureus 12S | AY043333 |
| Hapalemur griseus ala | AJ430037 |
| Hapalemur griseus griseus | AJ429211 |
| Hapalemur griseus griseus | AJ429210 |
| Hapalemur griseus meridionalis | AJ429206 |
| Hapalemur griseus meridionalis | AJ429205 |
| Hapalemur griseus occidentalis | AJ429212 |
| Hapalemur griseus occidentalis | AJ429214 |
| Hapalemur simus | AF175801 |
| Hapalemur simus | AF175800 |
| Indri indri | AY043340 |
| Indri indri | AJ429618 |
| Lemur catta | AF038013 |
| Lemur catta | AY012130 |
| Lemur catta | AF175779 |
| Lemur catta | AF175780 |
| Lepilemur dorsalis | AJ270676 |
| Lepilemur dorsalis | AJ270611 |
| Lepilemur edwardsi | AJ429621 |
| Lepilemur edwardsi | AY043346 |
| Lepilemur leucopus | AJ429622 |
| Lepilemur mustelinus | AJ429623 |
| Lepilemur mustelinus | AY585737 |
| Lepilemur mustelinus | AY585736 |
| Lepilemur ruficaudatus | AY043345 |
| Lepilemur ruficaudatus | AJ429624 |
| Lepilemur septentrionalis | AJ270629 |
| Lepilemur septentrionalis | AJ270660 |
| Loris lydekkerianus | AY773973 |
| Loris tardigradus | DQ073501 |
| Loris tardigradus | DQ073500 |
| Megaladapis edwardsi | AJ429626 |
| Microcebus griseorufus | AY582700 |
| Microcebus griseorufus | AY582699 |
| Microcebus jollyae | AY582686 |
| Microcebus jollyae | AY582684 |
| Microcebus jollyae | AY582687 |
| Microcebus mittermeieri | AY582677 |
| Microcebus mittermeieri | AY582680 |
| Microcebus murinus | AY582698 |
| Microcebus murinus | AY582697 |
| Microcebus ravelobensis | AY582672 |
| Microcebus ravelobensis | AY582674 |
| Microcebus rufus | AY582693 |
| Microcebus rufus | AY582692 |
| Microcebus sambiranensis | AY582676 |
| Microcebus simmonsi | AY582711 |
| Microcebus simmonsi | AY582703 |
| Mirza coquerelli | AJ429628 |
| Nycticebus coucang | DQ073504 |
| Nycticebus coucang | AJ309867 |
| Nycticebus pygmaeus | AY773976 |
| Otolemur crassicaudatus | DQ073506 |
| Otolemur crassicaudatus | DQ073505 |
| Otolemur garnettii | DQ073509 |
| Otolemur garnettii | DQ073508 |
| Otolemur monteiri argentatus | DQ073518 |
| Otolemur monteiri argentatus | DQ073517 |
| Otolemur monteiri argentatus | DQ073516 |
| Otolemur monteiri monteiri | DQ073515 |
| Otolemur monteiri monteiri | DQ073514 |
| Otolemur monteiri monteiri | DQ073513 |
| Perodicticus potto | AY773971 |
| Perodicticus potto | DQ073519 |
| Propithecus diadema | AY043341 |
| Propithecus diadema | AJ429619 |
| Propithecus tattersalli | AY043342 |
| Propithecus tattersalli | AF175794 |
| Propithecus tattersalli | AF175793 |
| Propithecus verreauxi | AJ429620 |
| Varecia variegata | AY043339 |
| Varecia variegata | AF175791 |

**Dataset 6: as in Dataset 5 but for a 333 bp subset of the 12S rRNA.**

This data minimizes the amount of missing data.

**Dataset 7: as in Dataset 6 but for a subset of the Lemuriformes.**

Lorisiformes were excluded to limit saturation effects and systematic errors associated. The tree was rooted on *Daubentonia madagascariensis*.

**Dataset 8: as in Dataset 7 but for a 197 bp subset of the 12S rRNA.**

This data minimizes even further the amount of missing data.

**Dataset 9: raw clustal alignment of a 372 bp 12S rRNA fragment.**

This alignment was generated with Clustal W 1.82 default parameters and Lorisiformes were excluded to limit saturation effects.

**Dataset 10: corresponds to the fusion of datasets 1 and 5 for 49 overlapping taxa.**

Note that when several haplotypes have been reported for a given species, we kept the one presenting the longest sequence length and exhibiting a minimal number of undetermined positions.

|  | [12S 985] | [cytb 1140] |
| --- | --- | --- |
| Archaeolemur edwardsii CH126 | NA | EU441939 |
| Archaeolemur majori CH146 | EU441938 | EU441940 |
| Arctocebus calabarensis | DQ073479 | AY441474 |
| Avahi laniger | AJ429616 | AY441453 |
| Avahi occidentalis | AY043343 | AY441454 |
| Cheirogaleus major | AY043347 | AY441457 |
| Daubentonia madagascariensis | AY043348 | U53569 |
| Eulemur coronatus | AY043335 | AY441448 |
| Eulemur fulvus | DQ073499 | U53576 |
| Eulemur macaco | AY043336 | AF081049 |
| Eulemur mongoz | AY043338 | AY441449 |
| Eulemur rubriventer | AY043337 | AF081052 |
| Euoticus elegantulus | DQ073480 | AY441469 |
| Galago alleni | DQ073522 | Z35095 |
| Galago gallarum | AF212950 | AF212970 |
| Galago moholi | DQ073481 | AF271410 |
| Galagoides demidoff | DQ073494 | AY441472 |
| Galagoides zanzibaricus | DQ073496 | AF212964 |
| Hadropithecus stenognathus CH421 | NA | EU441943 |
| Hapalemur aureus | AY043333 | AY441446 |
| Hapalemur griseus | AJ429211 | U53574 |
| Hapalemur simus | AF175801 | AJ428978 |
| Indri indri | AY043340 | AY441455 |
| Lemur catta | AF038013 | AF175960 |
| Lepilemur dorsalis | AJ270611 | DQ234886 |
| Lepilemur edwardsi | AY043346 | DQ109004 |
| Lepilemur leucopus | AJ429622 | DQ109007 |
| Lepilemur mustelinus | AY585737 | DQ109034 |
| Lepilemur ruficaudatus | AY043345 | DQ109017 |
| Lepilemur septentrionalis | AJ270660 | DQ234900 |
| Loris tardigradus | DQ073500 | AY441475 |
| Megaladapis edwardsi | AJ429626 | NA |
| Megaladapis sp | NA | AY894790 |
| Microcebus griseorufus | AY582700 | AY167075 |
| Microcebus murinus | AY582698 | AF285565 |
| Microcebus ravelobensis | AY582672 | AF285529 |
| Microcebus rufus | AY582693 | AF285552 |
| Microcebus sambiranensis | AY582676 | AF285556 |
| Mirza coquerelli | AJ429628 | DQ093175 |
| Nycticebus coucang | AJ309867 | NC_002765 |
| Nycticebus pygmaeus | AY773976 | AY687900 |
| Otolemur crassicaudatus | DQ073506 | AY441465 |
| Otolemur garnettii | DQ073508 | AF271412 |
| Palaeopropithecus sp | NA | AY894792 |
| Perodicticus potto | DQ073519 | AY441473 |
| Propithecus diadema | AY043341 | AY441452 |
| Propithecus tattersalli | AY043342 | U53573 |
| Propithecus verreauxi | AJ429620 | AY441451 |
| Varecia variegata | AY043339 | AY441450 |

**Dataset 11: corresponds to the fusion of datasets 2 and 6 for 49 overlapping taxa.**

Note that when several haplotypes have been reported for a given species, we kept the one presenting the longest sequence length and exhibiting a minimal number of undetermined positions.

|  | [12S 333] | [cytb 486] |
| --- | --- | --- |
| Archaeolemur edwardsii CH126 | NA | EU441939 |
| Archaeolemur majori CH146 | EU441938 | EU441940 |
| Arctocebus calabarensis | DQ073479 | AY441474 |
| Avahi laniger | AJ429616 | AY441453 |
| Avahi occidentalis | AY043343 | AY441454 |
| Cheirogaleus major | AY043347 | AY441457 |
| Daubentonia madagascariensis | AY043348 | U53569 |
| Eulemur coronatus | AY043335 | AY441448 |
| Eulemur fulvus | DQ073499 | U53576 |
| Eulemur macaco | AY043336 | AF081049 |
| Eulemur mongoz | AY043338 | AY441449 |
| Eulemur rubriventer | AY043337 | AF081052 |
| Euoticus elegantulus | DQ073480 | AY441469 |
| Galago alleni | DQ073522 | Z35095 |
| Galago gallarum | AF212950 | AF212970 |
| Galago moholi | DQ073481 | AF271410 |
| Galagoides demidoff | DQ073494 | AY441472 |
| Galagoides zanzibaricus | DQ073496 | AF212964 |
| Hadropithecus stenognathus CH421 | NA | EU441943 |
| Hapalemur aureus | AY043333 | AY441446 |
| Hapalemur griseus | AJ429211 | U53574 |
| Hapalemur simus | AF175801 | AJ428978 |
| Indri indri | AY043340 | AY441455 |
| Lemur catta | AF038013 | AF175960 |
| Lepilemur dorsalis | AJ270611 | DQ234886 |
| Lepilemur edwardsi | AY043346 | DQ109004 |
| Lepilemur leucopus | AJ429622 | DQ109007 |
| Lepilemur mustelinus | AY585737 | DQ109034 |
| Lepilemur ruficaudatus | AY043345 | DQ109017 |
| Lepilemur septentrionalis | AJ270660 | DQ234900 |
| Loris tardigradus | DQ073500 | AY441475 |
| Megaladapis edwardsi | AJ429626 | NA |
| Megaladapis sp | NA | AY894790 |
| Microcebus griseorufus | AY582700 | AY167075 |
| Microcebus murinus | AY582698 | AF285565 |
| Microcebus ravelobensis | AY582672 | AF285529 |
| Microcebus rufus | AY582693 | AF285552 |
| Microcebus sambiranensis | AY582676 | AF285556 |
| Mirza coquerelli | AJ429628 | DQ093175 |
| Nycticebus coucang | AJ309867 | NC_002765 |
| Nycticebus pygmaeus | AY773976 | AY687900 |
| Otolemur crassicaudatus | DQ073506 | AY441465 |
| Otolemur garnettii | DQ073508 | AF271412 |
| Palaeopropithecus sp | NA | AY894792 |
| Perodicticus potto | DQ073519 | AY441473 |
| Propithecus diadema | AY043341 | AY441452 |
| Propithecus tattersalli | AY043342 | U53573 |
| Propithecus verreauxi | AJ429620 | AY441451 |
| Varecia variegata | AY043339 | AY441450 |

**Dataset 12: corresponds to the fusion of datasets 3 and 7 for 36 overlapping taxa.**

Note that when several haplotypes have been reported for a given species, we kept the one presenting the longest sequence length and exhibiting a minimal number of undetermined positions.

|  | [12S 333] | [cytb 486] |
| --- | --- | --- |
| Archaeolemur edwardsii CH126 | NA | EU441939 |
| Archaeolemur majori CH146 | EU441938 | EU441940 |
| Avahi laniger | AJ429616 | AY441453 |
| Avahi occidentalis | AY043343 | AY441454 |
| Cheirogaleus major | AY043347 | AY441457 |
| Daubentonia madagascariensis | AY043348 | U53569 |
| Eulemur coronatus | AY043335 | AY441448 |
| Eulemur fulvus | DQ073499 | U53576 |
| Eulemur macaco | AY043336 | AF081049 |
| Eulemur mongoz | AY043338 | AY441449 |
| Eulemur rubriventer | AY043337 | AF081052 |
| Hadropithecus stenognathus CH421 | NA | EU441943 |
| Hapalemur aureus | AY043333 | AY441446 |
| Hapalemur griseus | AJ429211 | U53574 |
| Hapalemur simus | AF175801 | AJ428978 |
| Indri indri | AY043340 | AY441455 |
| Lemur catta | AF038013 | AF175960 |
| Lepilemur dorsalis | AJ270611 | DQ234886 |
| Lepilemur edwardsi | AY043346 | DQ109004 |
| Lepilemur leucopus | AJ429622 | DQ109007 |
| Lepilemur mustelinus | AY585737 | DQ109034 |
| Lepilemur ruficaudatus | AY043345 | DQ109017 |
| Lepilemur septentrionalis | AJ270660 | DQ234900 |
| Megaladapis edwardsi | AJ429626 | NA |
| Megaladapis sp | NA | AY894790 |
| Microcebus griseorufus | AY582700 | AY167075 |
| Microcebus murinus | AY582698 | AF285565 |
| Microcebus ravelobensis | AY582672 | AF285529 |
| Microcebus rufus | AY582693 | AF285552 |
| Microcebus sambiranensis | AY582676 | AF285556 |
| Mirza coquerelli | AJ429628 | DQ093175 |
| Palaeopropithecus sp | NA | AY894792 |
| Propithecus diadema | AY043341 | AY441452 |
| Propithecus tattersalli | AY043342 | U53573 |
| Propithecus verreauxi | AJ429620 | AY441451 |
| Varecia variegata | AY043339 | AY441450 |
